# PaLS Study of Sleep Deprivation and Mental Health Consequences of the COVID-19 Pandemic among University Students: A Cross-Sectional Survey

**DOI:** 10.3390/ijerph18189581

**Published:** 2021-09-11

**Authors:** Grzegorz Gruba, Przemysław Seweryn Kasiak, Joanna Gębarowska, Natalia Adamczyk, Zuzanna Sikora, Alicja Monika Jodczyk, Artur Mamcarz, Daniel Śliż

**Affiliations:** 1 Students’ Scientific Group of Lifestyle Medicine, 3rd Department of Internal Medicine and Cardiology, Medical University of Warsaw, 04-749 Warsaw, Poland; gebarowska.joanna@gmail.com (J.G.); adamczyk.natalia1997@gmail.com (N.A.); zznnsikora@gmail.com (Z.S.); alajodczyk4@gmail.com (A.M.J.); daniel.sliz@wum.edu.pl (D.Ś.); 2 Polish Society of Lifestyle Medicine, 00-388 Warsaw, Poland; artur.mamcarz@wum.edu.pl; 3 3rd Department of Internal Medicine and Cardiology, Medical University of Warsaw, 04-749 Warsaw, Poland; 4 School of Public Health, Postgraduate Medical Education Center, 01-813 Warsaw, Poland

**Keywords:** COVID-19, sleep quality, mental health, physical activity, blue light, screen time

## Abstract

The COVID-19 pandemic has changed the way many people live. To assess its impact on sleep quality and quantity, blue light exposure, and the mental health of Polish university students, a cross-sectional survey was conducted. Almost half of the participants were medical students (47.62%; *n* = 630). The majority of students were suffering from insomnia (58.13%, *n* = 769). Almost every third student was sleeping less than 7 h a day (30.39%, *n* = 402). Our study showed that a short sleep duration correlates with poorer mental health outcomes. Respondents who declared sadness and depression were more likely to suffer from insomnia (OR = 5.6997, 95% CI: 4.3641–7.4441). Difficulty with tasks was also more likely to co-occur with insomnia (OR = 5.4723, 95% CI: 4.3007 to 6.9630). The results of this study showed that the COVID-19 pandemic contributed to the deterioration of sleep quality and quantity as well as the psychological well-being of Polish students. It is important to take steps to promote proper sleeping habits to alleviate the risk of mental health disorders in this group of people.

## 1. Introduction

The outbreak of the COVID-19 pandemic has greatly changed the ordinary way many people function. Restrictions first introduced by the government in March 2020 to limit the spread of the pandemic forced people to limit social interactions. The increasing number of SARS-CoV-2 infections [1] made many people afraid of their own and their loved ones’ health. The unstable economic situation [2] has raised anxiety about the future. Based on observations and the available literature, it was assumed that the changes have affected people’s lifestyles, which could reflect on medical problems we will be dealing with in the future. Sleep disorders have been linked with increased sadness and decreased pleasure and interest in daily occasions [3]. Poor sleep quality and insomnia are associated with incident depression [4], insulin resistance, hypertension, diabetes, weight gain, stress [5], and they are connected with a tendency to drug dependence and tobacco and hypnotics use [6].

First reports show the devastating impact the SARS-CoV-2 pandemic has had on students. An assessment made on US university students showed that 71.26% felt their anxiety levels had risen since the start of the COVID-19 pandemic, and the majority were not able to manage that situation [7]. Authors of one of the first studies on the Polish population stated that university students were in need for help due to mental health issues [8]. Studies on adults point out that during the pandemic people noted a deterioration in physical activity (43%) and an increase in screen time (49%) and food consumption (34%) [9]. There was also a change in sleeping patterns [10]. In Greece, a high insomnia rate was reported [11].

To assess the lifestyle of Polish students, Pandemic against LifeStyle (PaLS), a cross-sectional online study, was established. It targeted the five most important aspects of everyday living: sleep, eating habits, physical activity, mental health, and drug usage. The aim of this article was to present the impact of the COVID-19 pandemic on the sleep quality and quantity, blue light exposure, and mental health of Polish university students.

## 2. Materials and Methods

A self-administered questionnaire consisting of six sections on demographics (age, sex, year of study, height, and weight), sleep, nutrition, physical activity, mental health, and drug use was created to conduct the study. A Polish version of the Athens Insomnia Scale (AIS) questionnaire [12,13] was used to assess insomnia prevalence. It is a well-established 8-item questionnaire, which is used in insomnia screening based on ICD-10 (10th revision of the International Statistical Classification of Diseases and Related Health Problems) criteria with 91% sensitivity and 87% specificity [14]. To measure sleep duration, three questions from Pittsburgh Sleep Quality Index were used, followed by one author’s question about time in front of a screen. Respondents’ physical activity was measured using the International Physical Activity Questionnaire. Mental health was assessed with 7 items from the Beck Depression Inventory (BDI). The sections on nutrition and drug use were composed of the authors’ questions. In the end of each section, participants were asked to mark how the pandemic has influenced their sleep, nutrition, physical activity, and mental health on the scale from −5 to +5 (“−5” meaning the most negative impact, “+5”—the most positive, and “0”—no influence).

Data was collected between 22 February and 3 April 2021. At that time in Poland, a lockdown was applied. Restaurants, pubs, cinemas, gyms, art galleries, and museums were closed; group meetings were prohibited; and classes took place in online or hybrid form (with only a small part in contact form). At this time, the number of infected people in Poland was ranging from 3890 on 22 February to 28,073 on 3 April, peaking at 35,251 on 1 April with the number of deaths at 17,621 and 749, respectively [15]. The target group of the survey was students of Polish universities. Respondents were reached via popular social media (Facebook and Instagram) and by email. The survey was anonymous, and responding to the survey was taken as consent to participate in the study.

**Figure 1:**
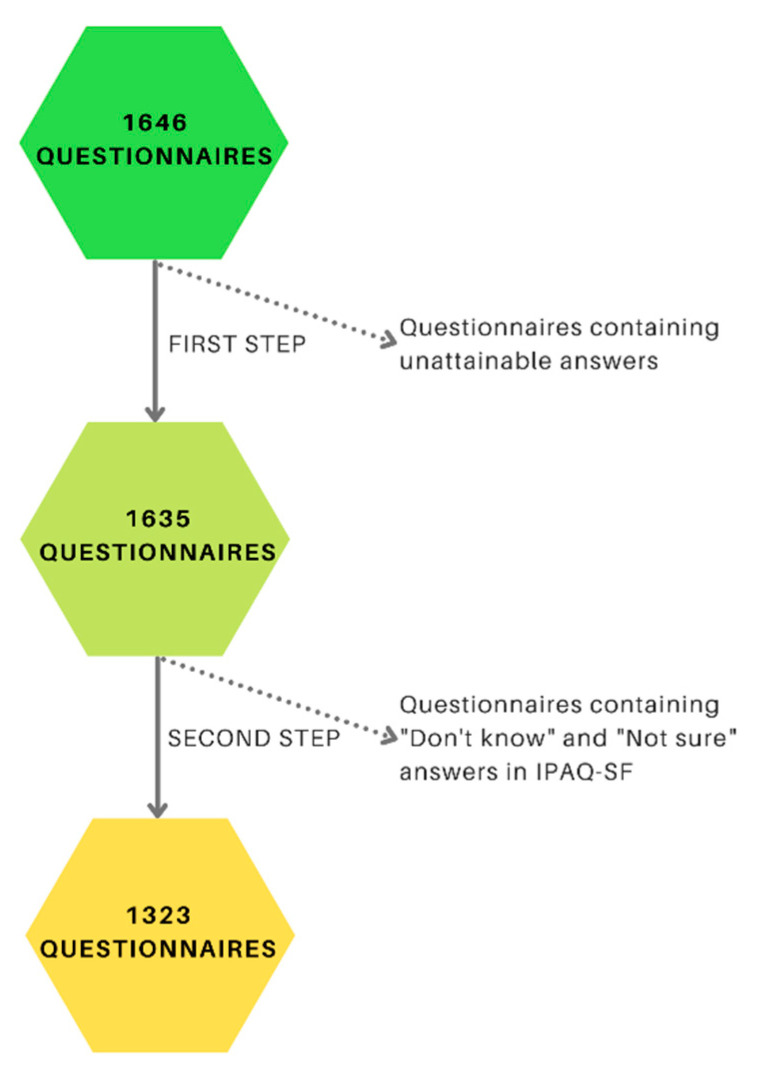
Data cleaning process.

The survey was filled by 1646 respondents. To raise the quality of the study, some questionnaires were excluded from analysis due to obtaining unattainable answers in which a high probability of untruth was predicted (e.g., BMI = 5 kg/m^2^, being physically active for 24 h per day, drinking 350 coffees/week etc; BMI—Body Mass Index.). Due to a scoring protocol of International Physical Activity Questionnaire- Short Form (IPAQ-SF), participants were excluded from the study because they chose either “don’t know” or “not sure” in those questions. The final number of responses included in the study was 1323 (Figure 1).

Statistical analysis was conducted in Statistica (StatSoft Inc., Kraków, Poland, version 13.3) and SPSS Statistics IBM, Armonk, NY, United States, version 27.0). Basic statistical calculations were made (normal distribution, mean, standard deviation). A chi-squared test was conducted to correlate insomnia, screen time, and mental health answers with other factors. Student’s *t*-test was introduced to establish correlations between insomnia and caffeine consumption. The statistical significance borderline was *p* < 0.05. Visual presentation of collected data was made in the form of a bar graph and a box plot.

## 3. Results

### 3.1. Sociodemographic Data

**Figure 2:**
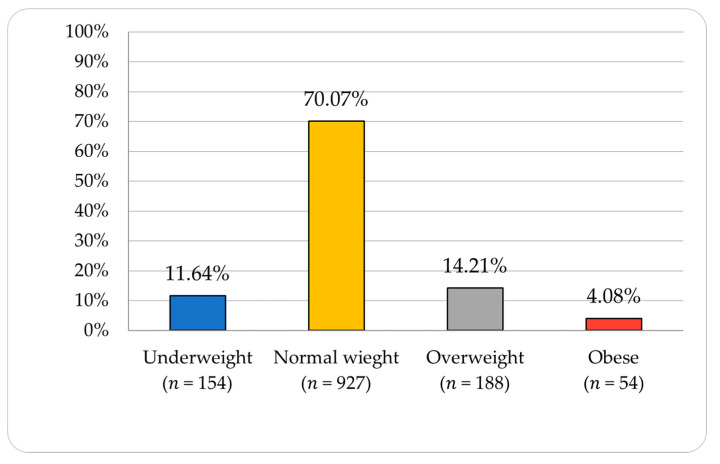
Weight distribution based on BMI (Body Mass Index).

Women represented 77.17% (*n* = 1021) of respondents. Men and women were not the only choice and thus “I prefer not to tell” was selected by 4 participants (0.30%), and “non-binary” was selected by 1 (0.08%). The mean age was 22.23 years old. Almost half of participants were medical students (47.62%; *n* = 630). Declared smokers were in the minority (16.63%; *n* = 220). Most of the students had a normal weight (70.07%; *n* = 927), but some of them were underweight (11.64%; *n* = 154), overweight (14.21%; *n* = 188), or obese (4.08%; *n* = 54) (Figure 2). The mean BMI was 22.27 kg/m^2^ ± 3.87. Most of the respondents were 1st-year students (33.40%; *n* = 442) and the least were 6th-year students (3.70%; *n* = 49).

### 3.2. Sleep Quality and Time in Front of the Screen

**Figure 3:**
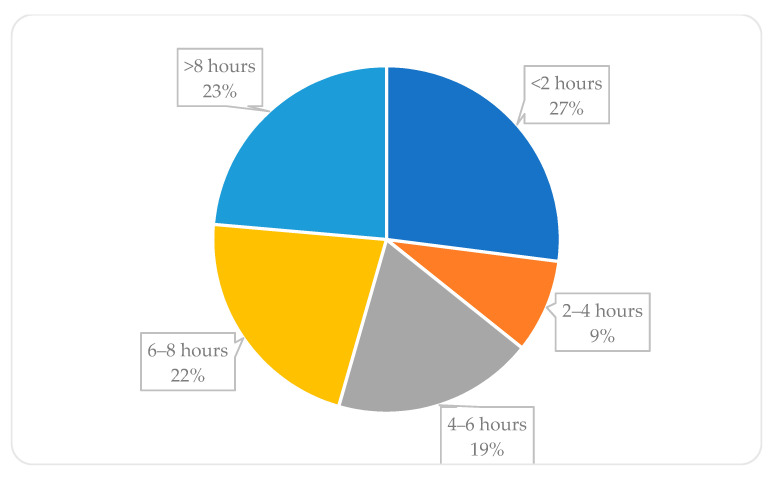
Time spent daily in front of blue-light-emitting devices.

The mean AIS score was 7.15 ± 4.25, differentiating visibly between men (6.04) and women (7.46). Most of the students were suffering from insomnia (58.13%, *n* = 769). Although insomnia prevalence varied between the years of study and types of study, it was not statistically significant for the year of study (*p* = 0.11111) and for the type of study (*p* = 0.36077). The median value of the going-to-bed time was 23 o’clock (24-h clock), and the median of the waking-up time was 8 o’clock. Sleeping 7 h a day was declared by 29.78% of students (*n* = 394), less than 7 h by 30.39% (*n* = 402), and more than 7 h by 39.83% (*n* = 527). Time spent daily in front of blue-light-emitting devices is shown in Figure 3. Almost one quarter (23%) spent more than 8 h a day in front of a screen.

### 3.3. Sleep-Disturbing Factors

**Figure 4:**
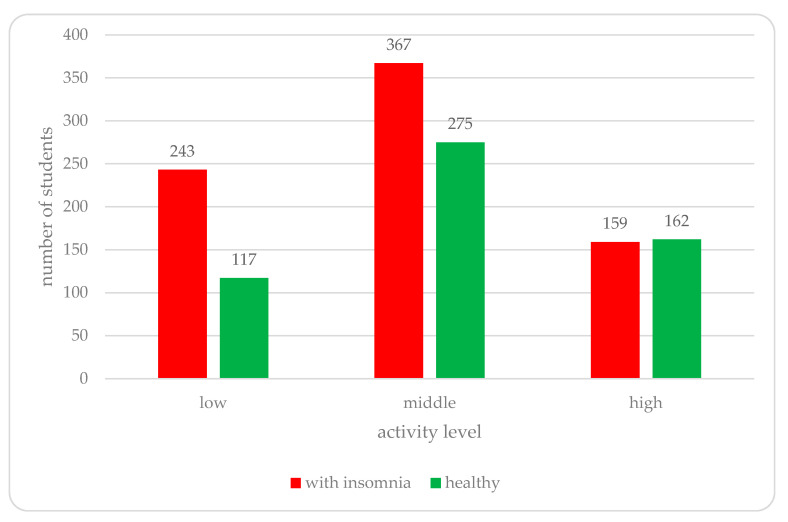
Insomnia prevalence in different activity levels.

The PaLS study results only confirmed that more blue light exposure can worsen one’s sleep quality, leading to insomnia (*p* = 0.02543). There were also other factors responsible for the higher insomnia rate. Respondents with insomnia drank higher amounts of coffee drinks per week (mean = 25.031) than respondents without insomnia (mean = 20.287, *p* = 0.000001). The results showed that students with low and middle activity levels had a higher chance of insomnia (*p* = 0.00001), as shown in Figure 4. Neither type of studies (*p* = 0.36077) nor the year of study (*p* = 0.11111) corresponded with insomnia, although sleep length varied between non-medical and medical students, who had a tendency to sleep less (*p* = 0.03545, OR = 1.2861, 95% CI: 1.0170–1.6264). Sex was another factor with an influence on insomnia. Women presented a higher chance of insomnia (*p* = 0.00018), but, surprisingly, there were no significant differences between genders when it came to sleep length (*p* = 0.07018).

### 3.4. Mental Health

**Figure 5:**
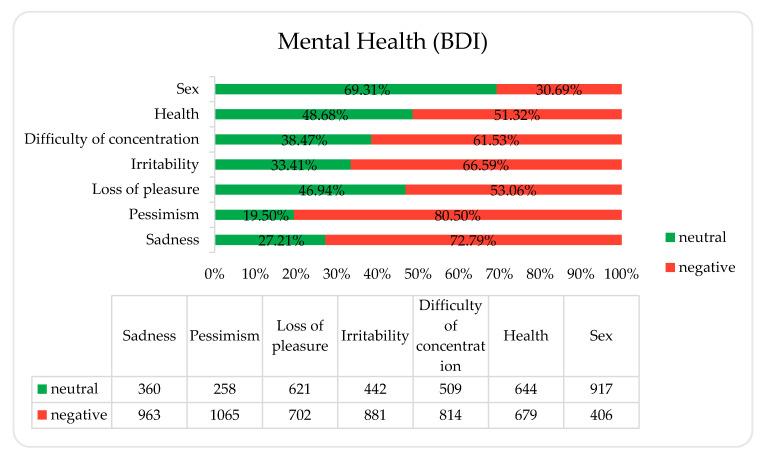
Answers to seven questions from the Beck Depression Inventory (BDI) divided into neutral and negative categories.

The percentage distribution of responses obtained from the mental health section are presented in Figure 5. Responses for each question were grouped into “neutral” if the respondent did not notice any deterioration in this area during the pandemic (responses that were scored 0 according to the Beck Depression Inventory) and “negative” if the respondent noticed any degree of deterioration caused by the pandemic (responses that were scored 1, 2, or 3 according to the BDI).

### 3.5. Mental Health Correlations

Respondents who declared sadness and depression in the mental health section were more than five times more likely to suffer from insomnia than those who did not feel sad or depressed (OR = 5.6997, 95% CI: 4.3641–7.4441, *p* = 0.0000). People who declared “being worried about the future” were 3.5 times more likely to suffer from insomnia compared to those who did not have such thoughts (OR = 3.5000, 95% CI: 2.6271–4.6627, *p* = 0.0000). Similarly, those who were worried about their health were also more likely to suffer from insomnia (OR = 2.5609, 95% CI: 2.0457–3.2059, *p* = 0.0000). People with insomnia were more likely to report that daily activities did not give them any pleasure (OR = 4.4539, 95% CI: 3.5254–5.6271, *p* = 0.0000). It is worth mentioning that medical students were less likely than non-medical students to report decreased reception of pleasure from daily activities (OR = 0.7259, 95% CI: 0.5844–0.9016, *p* = 0.00374). Respondents who reported feeling nervous were more likely to suffer from insomnia compared to those who did not (OR = 3.5007, 95% CI: 2.7572–4.4447, *p* = 0.0000). Difficulty with tasks was also more likely to co-occur in those with insomnia (OR = 5.4723, 95% CI: 4.3007–6.9630, *p* = 0.0000). Insomnia was also more common in people who reported a decreased interest in sex (OR = 2.2488, 95% CI: 1.7499–2.8900, *p* = 0.0000).

The PaLS study also showed that short sleep duration correlated with poorer mental health outcomes. People who slept less than 7 h a day were more likely to feel sad and depressed (OR = 1.7582, 95% CI: 1.3258–2.3315, *p* = 0.00008), worried about their future (OR = 1.4825, 95% CI: 1.0838 to 2.0280, *p* = 0.01338) and their health (OR = 1.5566, 95% CI: 1.2281–1.9730, *p* = 0.00024). Additionally, they were more likely to report that daily activities did not give them pleasure (OR = 1.8120, 95% CI: 1.4248–2.3043, *p* = 0.00000) and that they had difficulty completing tasks (OR = 1.7756, 95% CI: 1.3810–2.2829, *p* = 0.00001), while also being more likely to feel nervous (OR = 1.6039, 95% CI: 1.2376–2.0786, *p* = 0.00033) and to report a decreased interest in sex (OR = 1.6751, 95% CI: 1.3075–2.1460, *p* = 0.00004) compared to those who did not experience exposure to short sleep.

### 3.6. Results from Questions Subjectively Assessing the Impact of the Pandemic on Mental Health

**Figure 6:**
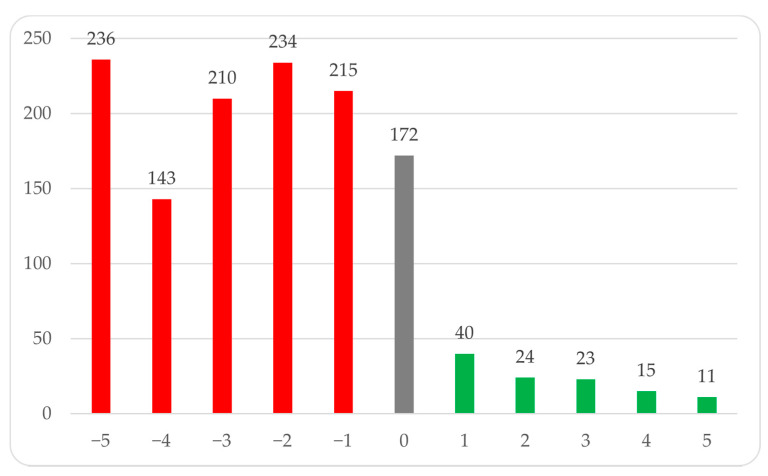
Respondents’ subjective assessment of the impact of the pandemic on mental health. Distribution of answers to the question.

In this section, the respondents subjectively rated on a scale of −5 to 5 the impact of the pandemic on their mental health. The majority of respondents reported a negative impact of the pandemic on their mental health (75.46%, *n* = 1038), while 13% (*n* = 172) observed no impact, and 8.54% (*n* = 113) reported a positive impact. The detailed distribution of responses is shown in the Figure 6.

### 3.7. Results from Questions Subjectively Assessing the Impact of the Pandemic on Sleep

**Figure 7:**
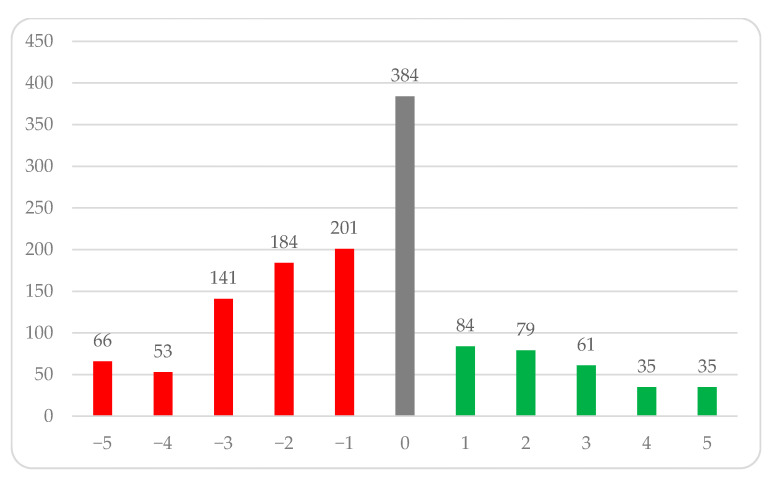
Respondents’ subjective assessment of the impact of the pandemic on sleep. Distribution of answers to the question.

In this section, respondents subjectively rated on a scale of −5 to 5 the impact of the pandemic on their sleep. Almost half of the respondents declared a negative impact of the pandemic on sleep (48.76%, *n* = 645), 29.02% (*n* = 384) observed neither a positive nor negative impact of the pandemic on sleep, while 22.22% (*n* = 294) rated the impact as positive. The detailed distribution of responses is shown in Figure 7.

## 4. Discussion

Good sleep quality can be crucial for students because it has a significant impact on learning performance [16]. The PaLS study indicated a 58.13% insomnia rate, which is higher than before the pandemic. In 2019, a study conducted on Polish young adults showed a 28.28% insomnia prevalence and a significant correlation between insomnia and depression symptoms. It has also been proven that females tend to have worse sleep quality [17]. Another study showed that insomnia was detected among 23% of Hungarian students [18] and 19.7% of Polish students (the only difference was that authors used a different cut-off score of 8 points, which, in the case of the PaLS study would result in a 42.03% insomnia prevalence), which was correlated with stress [19]. Based on the presented results, it can be deduced that the COVID-19 pandemic played a significant role in students’ sleep quality, but there were other groups of people, which could suffer even more during that hard time. In a study on Polish nurses, insomnia was observed among 47.8% of respondents [20]. Authors of another study indicated that 49% of Polish anaesthesiologists and intensivists were not satisfied with their sleep quality [21], and those numbers could have increased even higher during the pandemic due to the amount of work and stress medical workers were dealing with at a time.

Technology used close to bedtime is associated with poor sleep quality, and the more interactive the device (computers, smartphones, and gaming consoles), the more likely it is for users to have difficulties in falling asleep and to have unrefreshing sleep [22]. Mobile phone dependency and tablet use can lead to worse sleep quality [23]. A Polish study demonstrated that meeting screen time recommendations for adolescents reduced the odds ratio of adverse dietary behaviours [24]. An excessive amount of blue light can also have an impact on mental health. Results from an American study suggest that people who spend 4 to 6 h daily in front of the screen have almost a two times higher chance of being depressed (OR = 1.943), and it is even higher among those who spend more than 6 h in front of the screen (OR = 2.321) [25]. It proves that it is crucial to reduce blue light exposition to the minimum. The results from the PaLS study show a disturbing phenomenon with 64.26% of students exposed to blue light for more than 4 h a day. It can negatively influence not only their sleep quality but also their mental health. Another thought-provoking appearance is that more students have been using blue-light-emitting devices for more than 8 h a day (23%, *n* = 409) than sleeping more than 8 h a day (13%, *n* = 172)

The American Academy of Sleep Medicine and Sleep Research Society states that sleeping less than 7 h per night on a regular basis can have a negative impact on peoples’ health. It was linked with increased body weight and a higher prevalence of obesity, diabetes, hypertension, heart disease, and stroke. It also decreases immunological system functionality, increases pain, and is related to higher accident and death risk, as well as depression [26]. A higher mortality and short sleep correlation have also been confirmed [27]. The most frequent answer among students in the PaLS study when asked about sleeping time was the minimal recommended sleeping time for adults [26] (29.78%, *n* = 394, median = 7), but almost every third one did not meet those recommendations and was sleeping less than 7 h a day (30.39%, *n* = 402). The percentage of people sleeping less than 7 h varied among different authors, e.g., 31.7% and 56.7% of Uruguayan university students [28]; 36% of US university students [29]; 39.2% of Asian, African, and American (i.e., the Americas) university students [30]; 43.9% of Chinese university students [31]; and 50.2% of Iraqi medical students [3]. Results of these studies indicate that Polish students during the pandemic did not sleep too short in comparison with other studies. On average they slept longer; however, their sleep quality was still worse. It implicates that sleep quantity is not a major factor to look at when diagnosing sleeping problems. Moreover, it shows that during the pandemic there were fewer students with sleeping quantity problems.

The PaLS study supports the hypothesis that people who sleep less perform worse on mental health questionnaires [17]. The results we obtained show that restrictions introduced during the pandemic may contribute to the deterioration of the mental health of Polish students. In a study published in 2019 [32], 56.8% of respondents reported no sadness or depression, whereas, in the PaLS survey, this was only 27.21%. In the 2019 survey, 29.8% of students did not report concerns about the future, while, in our survey, it was 19.29%. What is more, in 2019, 70.6% of respondents reported taking pleasure in what they were doing, whereas less than half of our respondents (46.94%) enjoyed their activities. It is possible that this was due to stay-at-home regulations and cutting back on many of their usual activities. However, we did not ask our respondents what activities they enjoyed or why they perceived a possible deterioration in this area. In our survey, students reported being more nervous (66.59%) compared to the 2019 respondent group (40.6%). Among our respondents, 61.53% reported problems with concentration, whereas before the pandemic any deterioration in this area was reported by only 25.7%. Just over half of the respondents in our questionnaire were worried about their health in any way (51.32%), which is a higher percentage than before the pandemic, when such respondents were 24.6%. In the PaLS survey, as in the pre-pandemic survey, students rated their mental health best in the area of sexual interest (PaLS—69.31% neutral responses, 2019—82.1%), although, in the PaLS study, almost one third of them declared a deterioration in this aspect. This could be one of the most thought-provoking results, taking into consideration the fact that our respondents were all young people of a reproductive age.

## 5. Conclusions

Based on recent studies, it should be anticipated that the COVID-19 pandemic would leave a significant mark not only on the future public health but also on almost everyone in the world. However, in terms of mental health and sleeping habits, it could have done more damage to younger people, as it was assessed that the pandemic did not significantly affect older adults [10,33]. A high number of Polish students are at risk of insomnia and mental health disorders due to COVID-19-imposed restrictions. Moreover, most of them are aware of the negative impact the COVID-19 pandemic had on their sleep and psychological well-being. It is important for public health workers and physicians to be engaged in promoting techniques that enable students to cope with negative changes in mental health. What is more, they should inform undergraduates how to maintain proper sleeping habits. More studies in this area are needed for better assessment of the impact of pandemic-induced restrictions on sleep quality and mental health in the population of students. An in-depth analysis of these issues is necessary to evaluate and implement interventions.

## 6. Limitations

The PaLS study was conducted online. Therefore, we could not assure that all participants were university students. Respondents who filled the questionnaire were probably interested in the topic of a healthy lifestyle, so they could have better or worse results than other students. As the majority of respondents were female, there is a need to check the results with a larger male probe. Moreover, almost half of the respondents were medical university students, which could affect the outcome. Those disproportions could reflect on the results. Therefore, it might be necessary to conduct this study on a less-biased group. The survey did not include a question about the respondents having been ill or vaccinated against COVID-19, so it is not known whether this may have influenced the survey results. The survey was conducted during a relatively short time period. There is a chance that both sleep quality and mental health varied during the whole pandemic.

## Data Availability

The data presented in this study are available on request from the corresponding author. The data are not publicly available due to not obtaining consent from respondents for publishing the data.
